# What’s the cardiac rhythm at the time of cardiac arrest? Disputed dogma or true fact?

**DOI:** 10.1093/europace/euae299

**Published:** 2024-12-18

**Authors:** Philippe Maury, Nathan Marimpouy, Maxime Beneyto

**Affiliations:** Department of Cardiology, University Hospital Toulouse, 1 avenue Pr J Poulhès, Toulouse 31000, France; I2MC, INSERM UMR 1297, Toulouse, France; Department of Cardiology, University Hospital Toulouse, 1 avenue Pr J Poulhès, Toulouse 31000, France; Department of Cardiology, University Hospital Toulouse, 1 avenue Pr J Poulhès, Toulouse 31000, France; I2MC, INSERM UMR 1297, Toulouse, France

**Keywords:** Cardiac arrest, Sudden death, Ventricular fibrillation, Asystole, Pulseless activity

## Abstract

It was widely accepted that malignant ventricular arrhythmias (VA) are the main direct initial cause for cardiac arrest and sudden cardiac death (SCD), but diverging data tended to demonstrate that asystole or pulseless activity were becoming the most prevalent cardiac rhythms at the time of cardiac arrest. We challenge here these conceptions and reinforce the persisting prominent role of VA in SCD.

Sudden death is defined by an unexpected natural death occurring within < 1 h from the onset of symptoms (or within 24 h of last being seen alive when unwitnessed) in a person without any prior condition that would appear fatal at short term. Sudden death is mainly related to cardiac causes (« sudden cardiac death ») (SCD) after excluding less common vascular, neurological, or respiratory causes. SCD is (or was) usually accepted—for most physicians and for the majority of cases—as the final result of the occurrence of malignant VA—i.e. ventricular fibrillation/tachycardia (VT/VF)—degenerating into lethal cardiac arrest if not successfully reanimated.

Historically—and we all cardiologists have grown with this dogma—VT/VF were pointed to be responsible for most SCD. Forty years ago, Holter recordings at the time of 69 cases of SCD revealed that 59% were caused by VF and 19% by torsades de pointes, letting 22% resulting from asystole and a few cases from atrio-ventricular block.^1^

This widely accepted prominent causal role of VA in SCD was the rationale for developing implantable cardioverter defibrillators (ICD) and more recently wearable or semi-automated external defibrillators. This would not have been the case when other competing mechanisms of cardiac arrest had been felt to be relevant. In fact, medical history about the efficiency and the large deployment of such devices throughout the world further reinforce the major role of malignant VA as a major—if not the leader—mechanism leading to cardiac arrest and SCD, decreasing all-cause mortality, even in the presence of other causes of death.^2^

Although widely accepted by the medical—and especially cardiological—community, this dogma was however challenged by some, after the surprising findings of a low rate of shockable cardiac rhythms at the time of first ECG recording in subjects presenting with in-hospital^3,4^ or out-hospital^5,6^ cardiac arrests undergoing reanimation. Some further decline in the proportion of patients with a shockable initial rhythm was even shown,^7^ while this was also correlated to age.^6^ Pulseless activity or asystole were found much more frequently in these subjects, challenging the prominent role of VA as the major cause for SCD.

This is not trivial, since it dramatically changes the relevance of defibrillation and chances of survival. These data merit also to be challenged as well, because possibly related to special settings and not expandable to the whole spectrum of SCD, and cannot be considered demonstrating the true initial causes for SCD in our opinion.

First, in-hospital cardiac arrests are probably not representative of ‘common’ SCD, which is known to occur outside hospital in the majority of cases.^3^ Patients dying suddenly in hospital are probably sicker and often hospitalized because of advanced heart diseases or acute complications, thus more prone to pulseless activity or asystole which may represent the final evolution of end-stage cardiomyopathy.

Secondly, the rate of shockable rhythm is simply a function of the no-flow (delay before reanimation initiation) or low-flow (duration of initial reanimation) durations. Even if shockable rhythms were recently observed in only one-third of more than 50.000 out-of-hospital cardiac arrests, they were in fact present in ∼ 60% of patients undergoing the earliest resuscitation maneuvers (first minutes), then gradually lowered over time.^8^ The documented heart cardiac rhythm within 2 min from 106 out-of-hospital cardiac arrest was VF in 64%.^9^ In 14.065 out-of-hospital cardiac arrests, the first ECG showed VF in 43% of patients, but the incidence of VF at the time of cardiac arrest was estimated to be 60–70% in all patients and 80–85% of cases with heart disease.^10^ In 873 out-of-hospital cardiac arrests, it was shown that the probability of recording VF decreased and that of documenting asystole increased as time from collapse elapsed.^11^ The slow decrease over time in the proportion of VF after cardiac arret^10^ is probably following the deterioration of metabolic conditions. This also may explain the high rates of terminal pulseless activity or asystole sometimes mentioned in out-of-hospital cardiac arrests,^5,6^ as low flow/no flow time durations increase, further leading to unsuccessful reanimation. Alternatively, delayed reanimation may find patients with terminal low voltage VF, considered as a non-shockable rhythm^10,11^ and interpreted as apparent asystole, but in fact which is true VF. This still non-investigated part of the ‘non-shockable rhythms’ would likely change the ratio between VF and true asystole. All these arguments are reflected by recent data, which, in opposition to the above-mentioned works, show a majority of shockable rhythms at the time of out-of-hospital cardiac arrests.^4,12,13^

Thirdly, even if devices are implanted in selected populations prone to VA, SCD unrelated to VA are marginal in ICD patients,^14^ whereas asystole, pulseless electrical activity and respiratory arrests represented only 25% of sudden cardiac arrest in patients with wearable ICD.^15^ Initial shockable rhythm was demonstrated in 55% of memories of automated external defibrillators when systematically downloaded.^16^

In our own series of 310 successive patients referred for resuscitated SCD to our institution along the last 11 years, initial cardiac rhythm was ascertained from first ECG or monitor tracing or from data from semi-automatic external defibrillators. In coronary artery disease patients, first documented tracing showed VF in 68% (*Figure 1*), pulseless activity/asystole in 20%, while VT was documented in 12%. Initial cardiac rhythm was FV in 87% of non-coronary cases (unpublished data).

**Figure 1:**
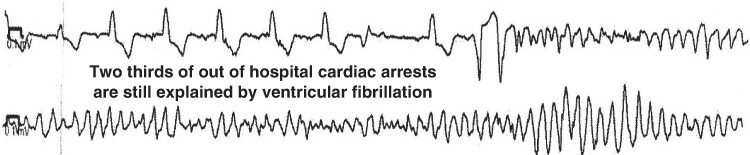
Exemple of ventricular fibrillation precipitating cardiac arrest and responsible for sudden cardiac death.

Thus, even if some doubt had been instilled over the past years, VA likely remain indeed the main direct initial cause for cardiac arrest and SCD, at least when occurring outside the hospital. Any preventive and therapeutic measures should still be directed towards VA in the future.

## Data Availability

Data available on demand at mauryjphil@hotmail.com.
